# Mechanisms of the regulation of ester balance between oxidation and esterification in aged Baijiu

**DOI:** 10.1038/s41598-020-74423-z

**Published:** 2020-10-13

**Authors:** Yonghui Deng, Ayuan Xiong, Kun Zhao, Yaru Hu, Bisheng Kuang, Xiang Xiong, Zhilong Yang, Yougui Yu, Qing Zheng

**Affiliations:** 1 School of Food and Chemical Engineering, Shaoyang University, Shaoyang, 422000 China; 2 Xiangjiao Institute for Liquor Engineering, Shaoyang University, Shaoyang, 422000 China; 3 Hunan Province Key Laboratory of New Technology and Application of Ecological Baijiu Production, Shaoyang University, Shaoyang, 422000 China

**Keywords:** Analytical chemistry, Physical chemistry

## Abstract

The influence of oxidation and esterification on the ester balance of aged Baijiu and methodology for the rational design of liquor flavors to optimize the practice of Baijiu production are not completely understood. We investigated the influence of ester balance on the flavor compounds of aged Baijiu by conducting constituent analysis of Baijiu from different aging times (0, 1, 2, 3, 4, 5, and 10 years). The changes of the main flavor compounds in the aging process were determined, and the correlations among different chemical reactions, such as oxidation, hydrolysis, and esterification, were systematically expounded. Furthermore, cluster analysis of the heat map indicated significant differences between aged Baijiu and new Baijiu and recommended a suitable aging time of 2–3 years.

## Introduction

Baijiu is a typical Chinese liquor originating from the Western Han Dynasty, which plays an important role in practical life in China and constitutes a unique position in the liquor industry with an annual production value of approximately 52 billion dollars^1,2^. In recent decades, with the wide application of chromatographic analysis technology, the chemistry behind Baijiu brewing has developed rapidly, providing the scientific basis for Baijiu production^3,4^. However, the production process in practice is not fully established because many factors influence Baijiu production^5^. The differences in raw materials, fermentation, distillation, aging, and other processes will have a direct effect on product quality, leading to product governance instability, which hinders the development of the Baijiu industry^6–8^.

On the basis of the differences in brewing materials and brewing techniques, Baijiu can be distinguished through its aroma, i.e., its main aroma components^9^. Thus far, there are 11 kinds of Baijiu^10^. For example, the Luzhou-flavor Baijiu is characterized by the mixed aroma of ethyl acetate and ethyl caproate^11^. The Maotai-flavor Baijiu is characterized by the mixed aroma of ethyl acetate and ethyl butyrate^12^. Notably, aging is an essential and crucial step in the formation of the Baijiu flavor (Baijiu without aging is pungent and acrid)^13–16^. Acid and ester compounds can be balanced during the aging process through different chemical reactions, such as oxidation, esterification, and hydrolysis. Wei Jia et al. reported that ester compounds, such as hexyl fumarate and butyl malonate, increase slowly through esterification and reach their maximum value in a 17-year-old Baijiu^2^. Lin Zhu et al. reported a positive correlation between acids and esters (i.e., hexanoic and acetic acids) and between alcohols and esters (i.e., 2-phenyl-1-ethanol) after 1 year of aging, which can be attributed to esterification^13^. However, most of the previous studies focused on one or more specific reactions and failed to determine the correlations among different chemical reactions. At present, the chemical reaction mechanism of aging has not been determined both at home and abroad, thereby needing further verification through systematic studies of the aging theory.

Therefore, in this study, the contents of the main flavor compounds in Baijiu samples of different aging years were determined by gas chromatography, and the correlations among different chemical reactions, such as oxidation and esterification, were systematically expounded. Oxidation, which is caused by the dissolved oxygen, converts alcohol into the corresponding acid that reacts with ethanol to form the corresponding ester. Thus, oxidation is the cause of the increase in ester content during aging. However, at the same time, if the content of a certain ester in the new Baijiu is high, the ester will undergo volatilization and hydrolysis, and the amount of the ester will be reduced so that a balanced esterification reaction can be achieved. If the content of a certain ester in the new Baijiu is low, then oxidation increases the content of ester substances so that a balanced esterification can be achieved. This study determines the correlations among different chemical reactions in aged Baijiu, which can be utilized by the food community as a reference to reveal the chemistry behind Baijiu or any distilled spirit.

## Materials and methods

### Baijiu samples

The Baijiu samples were collected from the manufacturer Xiangjiao Group Ltd., Shaoyang, China.

Baijiu samples were aged for 7 different years (fresh Baijiu as 0-year aged Baijiu, 1-year aged, 2-year aged, 3-year aged, 4-year aged, 5-year aged, and 10-year aged Baijiu). The mention of Baijiu brand name does not mean any research contact with the manufacturer, nor is the mention for advertising. All Baijiu samples used in this study were directly collected from storage containers without any additive.

### Methods

Identification and quantification of flavor compounds in Baijiu were performed by Agilent 7890B gas chromatography (Agilent Technologies Co. Ltd.), shown in Supplementary Tab. S1, and Supplementary Fig. S1–S7 in supporting information. 2-ethylbutyric acid and n-pentyl acetate were used as the internal standard and the concentrations were 18.122 and 17.316 mg/100 ml, respectively. Baijiu samples (4.9 ml) were spiked with 0.1 ml internal standard. The analytical capillary column was an Agilent CP-Wax 57 CB column (0.25 mm × 50 m × 0.2 μm) with a flow rate of 1.0 mL/min. The temperature program was as follows: oven temperature was held at 40 °C for 5 min, then raised to 50 °C at a rate of 3 °C/min and held for 6.5 min, raised at 6 °C/min up to 90 °C and held for 5 min, then raised to 130 °C at a rate of 10 °C/min and held for 2 min, then raised to 190° C at a rate of 5 °C/min and held for 1.4 min, finally raised to 195° C at a rate of 10° C/min and held for 20 min. The chemical standards for the flavor compounds were purchased from Merck (Germany). The qualitative analysis of targeted components was made by comparing the retention times between these compounds and the chemical standards, and the internal calibration curve was constructed for each compound measured using the standards^17^.

### Statistical analysis of experimental data

The reported result is the mean value of triplicate measurements and the data are expressed as the means of triplicate analysis (± S.E.M). Statistical analysis was performed with Origin 2019 (OriginLab Co., Northampton, USA).

## Results and discussion

**Figure 1:**
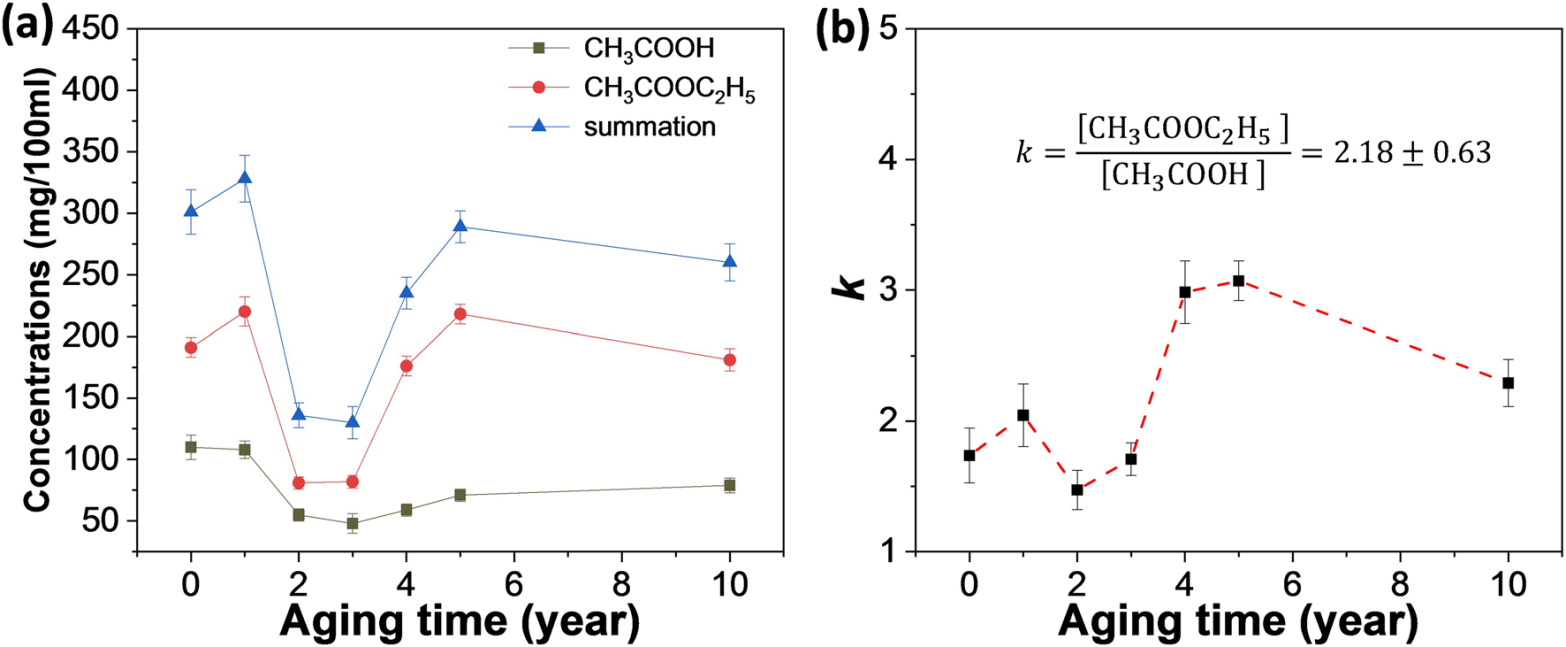
**(a)** The contents of ethyl acetate, acetic acid and the summation of ethyl acetate, acetic acid in seven liquors of different aging years (0, 1, 2, 3, 4, 5, and 10 years). **(b)** The *k* values in Eq. (2) measured for the liquor samples of different aging years.

The flavorist has carried on systematic research to Baijiu flavor type, it is generally accepted that the Baijiu aroma primarily comes from the ester^18,19^, such as ethyl acetate. In order to explore the effects of aging on the liquor quality, the contents of ethyl acetate and acetic acid in seven liquors of different aging years were tested. As shown in Fig. 1a (line in red), the content of ethyl acetate in 0-year and 1-year liquor sample stay comparatively high, reaching 232 mg/100 ml. Its content decreased at the early stage (2–3 years) and then increased until it reached a steady state (5–10 years) that resulting from the ester balance. The content of acetic acid (line in black in Fig. 1a) was lower than that of ethyl acetate, but the variation tendency of the two was the same. As shown in Eq. (1), acetic acid and ethanol esterifies to form the ethyl acetate. In liquor samples, the content of ethanol and water was > 50 times higher than that of acetic acid and ethyl acetate. Therefore, the equilibrium constant (*k*) of the reaction could be written as Eq. (2).

1 $${\text{CH}}_{{3}} {\text{COOH }} + {\text{ C}}_{{2}} {\text{H}}_{{5}} {\text{OH}} \rightleftharpoons {\text{CH}}_{{3}} {\text{COOC}}_{{2}} {\text{H}}_{{5}} + {\text{ H}}_{{2}} {\text{O}}$$

2 $$k=\frac{\left[{\mathrm{CH}}_{3}{\mathrm{COOC}}_{2}{\mathrm{H}}_{5} \right]}{\left[{\mathrm{CH}}_{3}\mathrm{COOH}\right]}$$

However, the liquor sample contains thousands of water-soluble compounds, *k* may be affected by the side reactions of Eq. (1)^20^. This concern can be counted by measuring the *k* value. The *k* values measured for the liquor samples of different aging years, were shown in Fig. 1b. As seen, *k* values of liquors from different aging years vary in a very narrow range [1.4, 3.2], with an average *k* value of 2.18 ± 0.63, which indicates that the side reactions in liquors does not have a significant effect on equilibrium constant in Eq. (2). Thus, it can be concluded that the content of acetic acid in the sample is directly correlated with the content of ethyl acetate.

In the process of liquor fermentation, microorganisms can not only produce a large amount of ethanol, but also use biological enzymes to oxidize ethanol to acetaldehyde, further oxidize to acetic acid, and finally obtain ethyl acetate through esterification^21^. Ethyl acetate, as a small molecule containing only four carbon atoms, has a low boiling point. As a result, the new distilled liquor contains a large amount of ethyl acetate (232 mg/100 mL), which rapid decreases with aging for 2–3 years. The rapid decrease can be mainly associated with the volatilization of ethyl acetate in addition to the partial hydrolysis of ethyl acetate. The end result is that the amount of ethyl acetate is reduced so that a balanced esterification reaction can be achieved.

Although acetic acid and ethyl acetate vary with each other with the equilibrium of esterification, their total contents can indicate the change of acetic acid during aging according to the law of conservation of mass. As shown in Fig. 1a (line in blue), after aging for 2–3 years, the esterification reached equilibrium and the total content was at a low level (134 mg/100 ml). After that, the total content presented a trend of slow increase. The results suggest that the oxidation caused by dissolved oxygen^13^ gradually becomes obvious after the new liquor has been aged for 2–3 years, which leads to the slow increase of acetic acid content. In other words, even the chemical reactions (e.g., oxidation and esterification) are cross-linked, the contribution of a certain chemical reaction to the compound contents is significantly different in aging periods.

**Figure 2:**
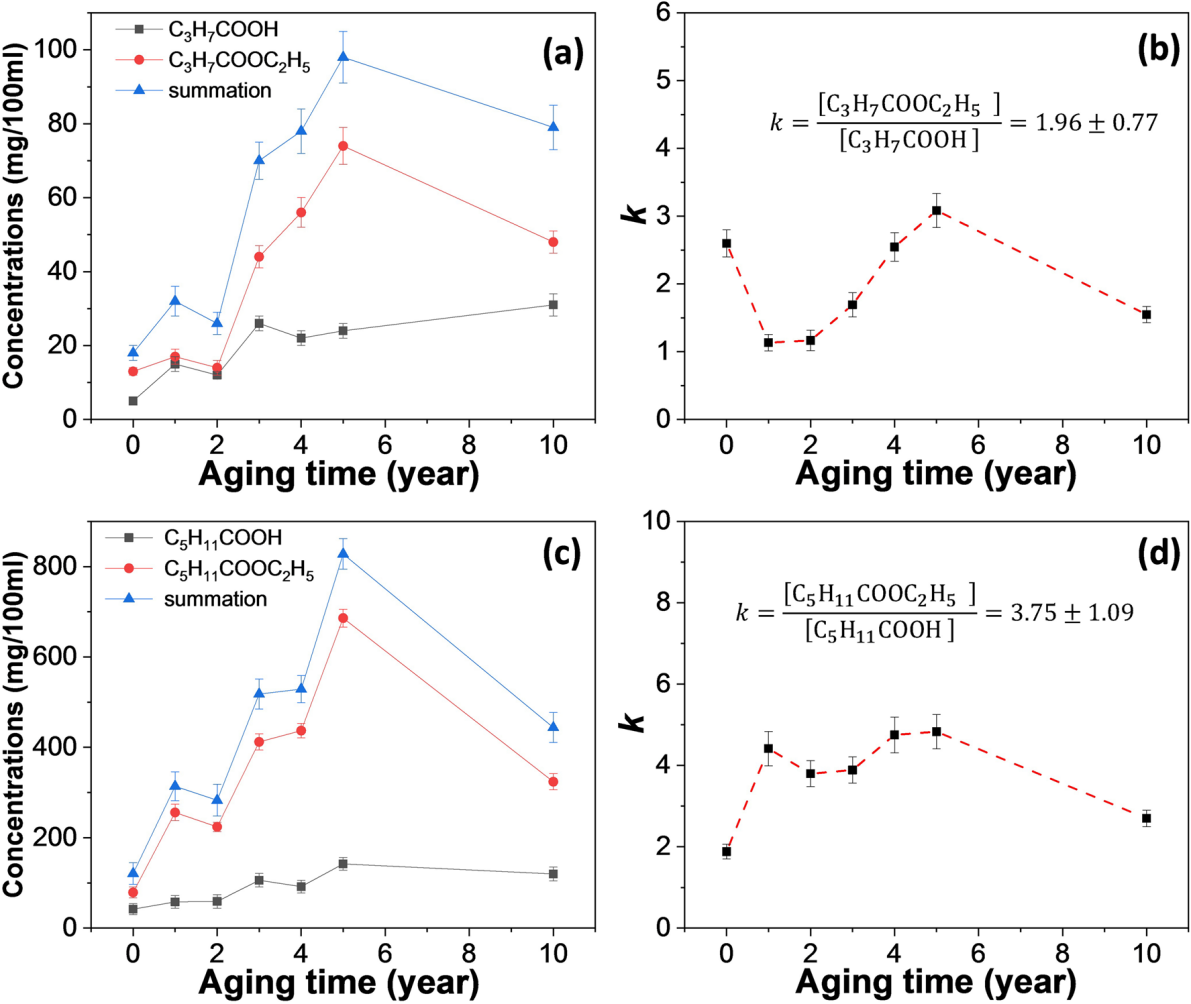
**(a)** The contents of ethyl butyrate, butyric acid and the summation of ethyl butyrate, butyric acid in seven liquors of different aging years. **(b)** The corresponding *k* values (shown in the insert) measured for the liquor samples. **(c)** The contents of ethyl caproate, caproic acid and the summation of ethyl caproate, caproic acid in seven liquors of different aging years. **(d)** The corresponding *k* values (shown in the insert) measured for the liquor samples.

The bouquet is derived from the ester-based complex aroma. Besides ethyl acetate, ethyl butyrate and ethyl caproate are also important sources of Baijiu flavor (e.g., Luzhou-flavor liquor). Similar to the above experiments, the contents of ethyl butyrate/butyric acid and ethyl caproate/caproic acid in seven liquors of different aging years were tested. As shown in Fig. 2a, the content of ethyl butyrate and butyric acid increased at the early stage (0–5 years) until it reached a steady state. The content of ethyl butyrate reached the highest level in the Baijiu sample aged for 5 years, reaching 71 mg/100 ml. The corresponding equilibrium constant *k* value is in the range of [1.1, 3.1], and the mean value is 1.96 ± 0.77, shown in Fig. 2b. As shown in Fig. 2c, the contents of ethyl caproate and caproic acid also increased at the early stage (0–5 years) until it reached a steady state. The content of ethyl caproate was the highest in the Baijiu sample aged for 5 years, reaching 680 mg/100 ml. The corresponding equilibrium constant *k* value is in the range of [1.9, 4.8], with an average value of 3.75 ± 1.09, shown in Fig. 2d. It can be seen that both ethyl butyrate and butyric acid, as well as ethyl caproate and caproic acid, are correlated with each other, indicating that the reversible equilibrium of esterification is an important factor in the content of corresponding acid and ester. At the same time, the contents of ethyl butyrate and ethyl caproate, display a slow increasing trend in the aging process. These results suggest that the oxidation caused by dissolved oxygen may be an important factor that related with the content of ester compounds during aging.

**Figure 3:**
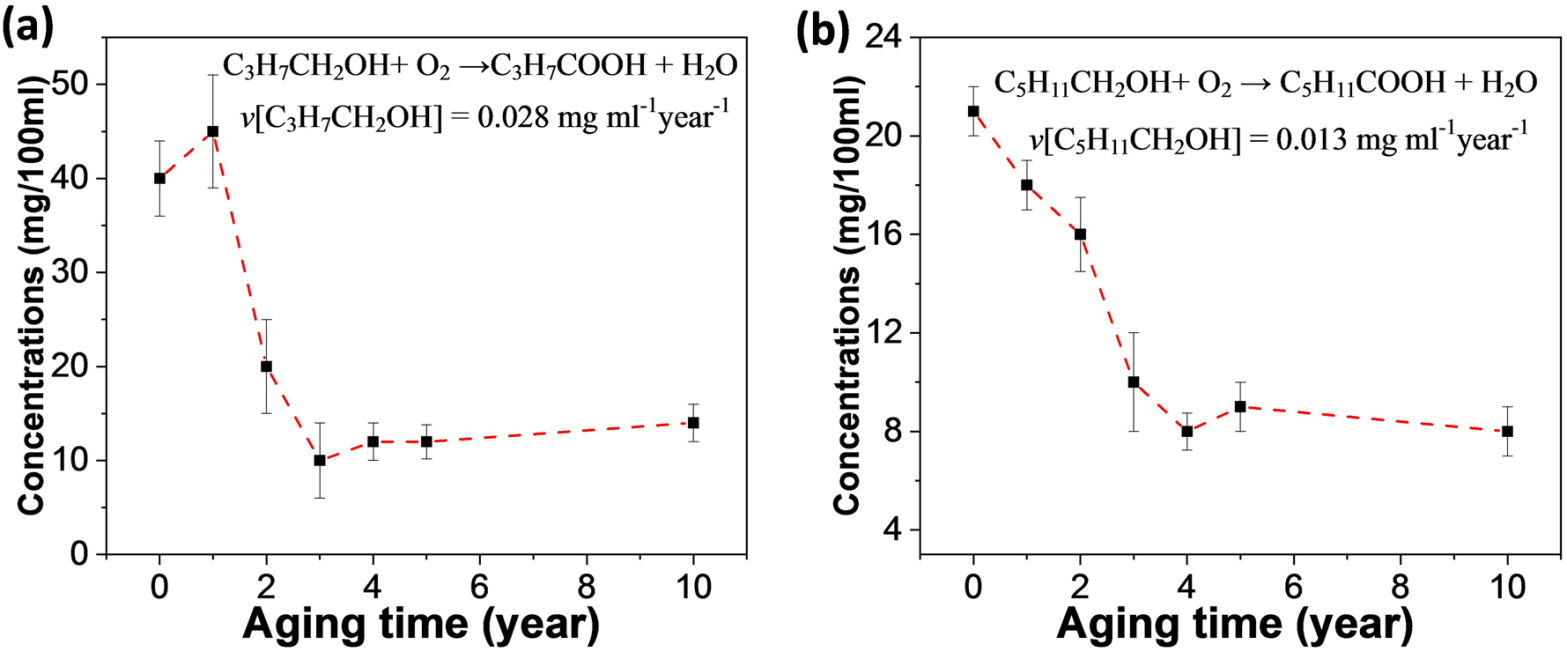
The contents of butanol **(a)** and hexanol **(b)** in seven liquors of different aging years. The consumption rates of butanol and hexanol are shown in the insert.

In order to explore the effect of oxidation on aging, the contents of butanol and hexanol in seven liquors of different aging years were tested. As shown in Fig. 3a, the content of butanol decreased rapidly at the early stage (0–3 years) until it reached a steady state. The butanol content in the new liquor was 41 mg/100 ml, and after three years of aging, the butanol content decreased to 11 mg/100 ml. It can be seen that the oxidation of butanol by dissolved oxygen in new liquor is very obvious. In addition, the content of butyraldehyde in the liquor sample is at a very low level (< 1 mg/100 ml), so the oxidation process of butanol can be written as Eq. (3), and the consumption rate of butanol is shown in the insert in Fig. 3a.

3 $${\text{C}}_{{3}} {\text{H}}_{{7}} {\text{CH}}_{{2}} {\text{OH}}\, + \,{\text{O}}_{{2}} \, \to \,{\text{C}}_{{3}} {\text{H}}_{{7}} {\text{COOH}}\, + \,{\text{H}}_{{2}} {\text{O}}$$

As shown, the consumption rate of butanol in the aging process of the first three years was 0.028 mg ml^−1^ year^−1^. The containers used to store liquor samples during aging are earthenware made of natural clay, which is characterized by a slight breathability^22^. Therefore, during the long aging process, enough oxygen from the outside enters the interior of the pottery to make up for the oxygen consumed by the oxidation reaction, ensuring that the dissolved oxygen remains saturated. As can be seen from Fig. 1a, ethyl acetate has a high content in new liquor. However, the content of ethyl butyrate in the new liquor is low as butanol produced by microorganisms in the fermentation process is very limited. Therefore, the liquor can only rely on the aging process to oxidize butanol to form butyric acid through the slow oxidation by dissolved oxygen, so as to increase the content of ethyl butyrate.

As shown in Fig. 3b, the content of hexanol decreased rapidly at the early stage (0–3 years) until it reached a steady state. The content of hexanol in the new liquor was 21 mg/100 ml. After three years of aging, the content of hexanol decreased to 9 mg/100 ml, and the consumption rate of hexanol in the first three years of aging was 0.013 mg ml^−1^ year^−1^. The dissolved oxygen oxidizes hexanol to caproic acid, which is then converted to ethyl caproate by esterification, so as to increase the content of ethyl caproate in the liquor sample. The above results indicated that both butanol and hexanol were converted into corresponding ester compounds by dissolved oxygen during aging. In Fig. 2a,c, it can be seen that the contents of butyric acid and caproic acid increase slowly in the aging process, which corresponds to the decrease of butanol and caproic alcohol.

**Figure 4:**
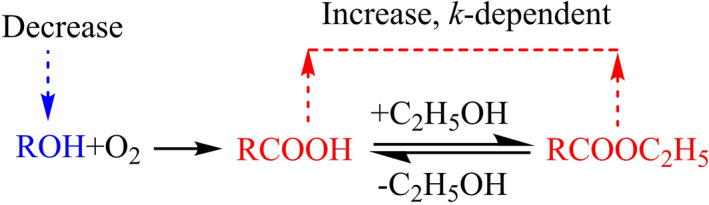
Schematic representation of the correlation among the chemical reactions in the aged Baijiu.

The above results indicated that the alcohols, acids and esters in the liquor had a certain correlation in the aging process. As shown in the scheme (Fig. 4), the oxidation caused by dissolved oxygen converts alcohols into corresponding acids, which react with ethanol to produce corresponding esters. On the one hand, oxidation is the cause of ester content increase during aging. On the other hand, the esterification reaction between acid and alcohol is reversible: (1) If the ester content in the new liquor is high (e.g., ethyl acetate), the ester will undergo volatilization and hydrolysis, and the amount of ester will be reduced so that the esterification reaction can reach a balance. It should be noted that this hydrolysis process is accompanied by oxidation, however, the reaction rate of oxidation is comparatively slow and is "masked" by the hydrolysis reaction; (2) If the ester content in the new liquor is low (e.g., ethyl butyrate and ethyl caproate), the oxidation makes the ester content increase, so that the esterification to reach a balance.

**Figure 5:**
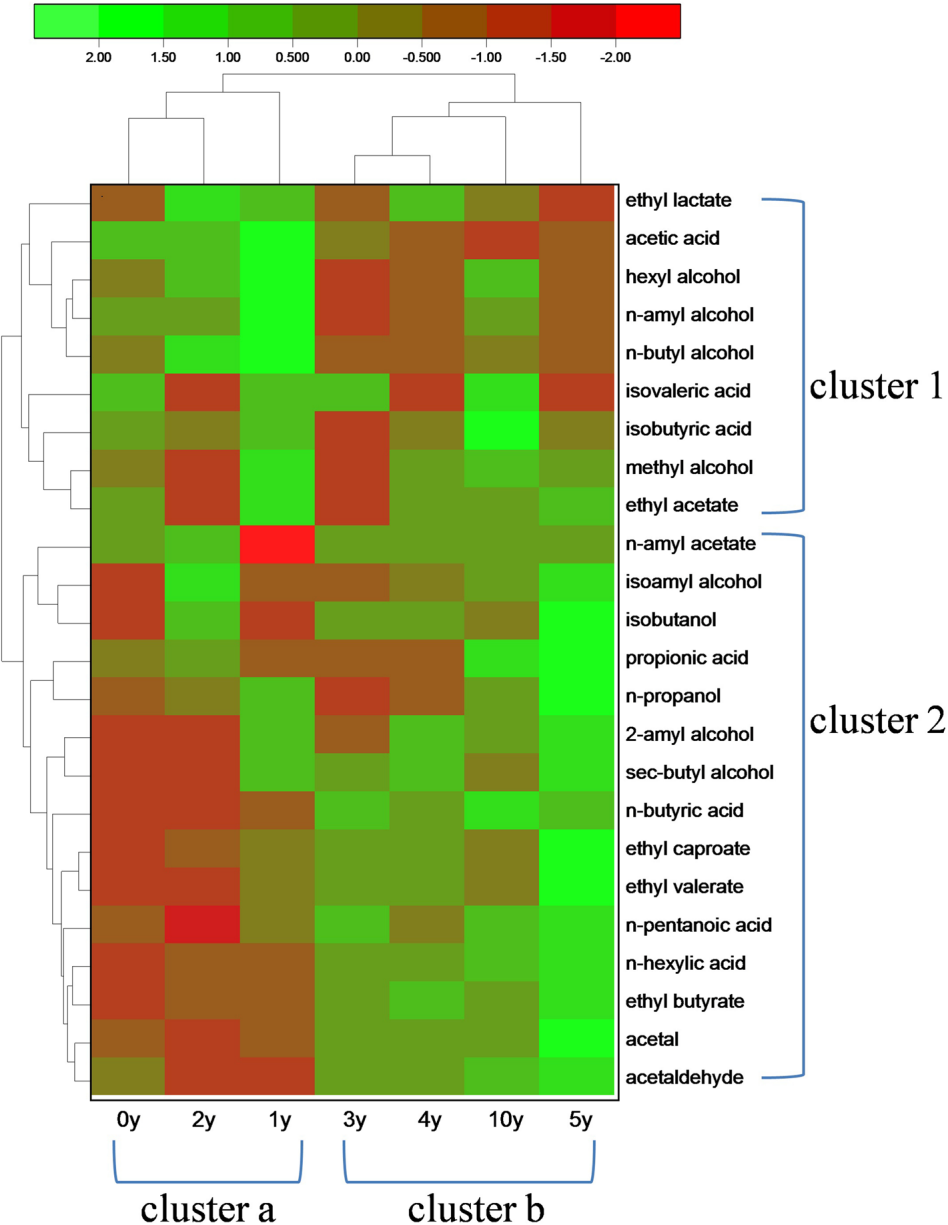
The main compounds in Baijiu showed different trends of variation during aging: A heat map of 24 compound contents in Baijiu during the 10-year aging process.

The change in the contents of characteristic compounds not only indicates the chemical mechanism of aging process, but also indicates the taxonomic distances and evolutionary relationships between liquors from different aging time, since the compounds are related to each other. Therefore, a phylogenetic tree was deduced based on the constituent analysis of Baijiu from different aging times. A total of 24 flavor compounds were selected and detected for the reasons: (1) compounds that hold a comparatively higher concentration, (2) compounds that contributed to the main aroma and flavor, and (3) compounds related to the safety indicators according to national standards of Baijiu. As shown in Fig. 5, changes in contents of these compounds can be chiefly divided into two groups based on the cluster analysis of the heat map, which visually reflects the contents of the 24 compounds: the compounds in the two clusters showed similar trends to those of *n*-butyl alcohol (cluster 1, Fig. 3a), and ethyl butyrate (cluster 2, Fig. 2a). More importantly, cluster analysis in the aging time shows that the liquors can be divided into two groups: 0 year, 1 year, and 2 years (cluster a); 3 years, 4 years, 5 years, and 10 years (cluster b). The results of cluster analysis indicated that there were significant differences between the aged liquor samples and the new liquor samples. The time required for aging is related to the storage environment of the liquor sample. And the concentration of dissolved oxygen and ambient temperature are the most important factors affecting the aging time. According to the result, the suitable aging time of the liquor samples studied in this paper is 2–3 years. Meanwhile, this result also verified the aging mechanism mentioned above, as shown in Fig. 4.

## Conclusions

In summary, we determined the correlation between alcohols, acids, and esters in the aging process of Baijiu by conducting constituent analysis of Baijiu from different aging times (i.e., 0, 1, 2, 3, 4, 5, and 10 years). Notably, oxidation induces the increase in ester content during aging by converting alcohols into the corresponding acids and esters. However, the esterification reaction between acid and alcohol is reversible: (1) if the ester content (e.g., ethyl acetate) in the new liquor is high, then it will undergo volatilization and hydrolysis and its amount will be reduced so that a balanced esterification reaction can be achieved and (2) if the ester content (e.g., ethyl butyrate and ethyl caproate) in the new liquor is low, then oxidation increases the ester content so that a balanced esterification reaction can be achieved. Moreover, this study observed certain compound differences within aged Baijiu, which can be utilized by the food community as a reference to reveal the chemistry behind Baijiu or any distilled spirit.

## Supplementary information

Supplementary Information.
