# Supplementary material for: Mechanisms of the regulation of ester balance between oxidation and esterification in aged Baijiu
Source: Sci Rep. 2020 Oct 13;10:17169. doi: 10.1038/s41598-020-74423-z (PMC7555894; doi:10.1038/s41598-020-74423-z)
Supplement: Supplementary file 1 — Supplementary Information. [file 41598_2020_74423_MOESM1_ESM.pdf]

Supporting information

Mechanisms of the regulation of ester balance between oxidation and esterification in aged Baijiu

Yonghui Deng [a], Ayuan Xiong[a], Kun Zhao[a], Yaru Hu[a], Bisheng Kuang[b], Xiang Xiong[b], Zhilong Yang[b], Yougui Yu[a,b,c,*], and Qing Zheng[a,b,c,*]

[a]*School of Food and Chemical Engineering, Shaoyang University, Shaoyang 422000, China*

[b]*Xiangjiao Institute for Liquor Engineering, Shaoyang University, Shaoyang 422000, China*

[c]*Hunan Province Key Laboratory of New technology and Application of ecological Baijiu production, Shaoyang University, Shaoyang 422000, China*

---

*Corresponding author:

qz@hnsyu.edu.cn (Q. Zheng); 648707465@qq.com (Y. Yu)

School of Food and Chemical Engineering, Shaoyang University, Shaoyang 422000, China

Fax: +86 739 5432250

Tel: +86 18910506824

Table S1. The constituent analysis of Liquor samples performed by gas chromatography. The concentration unit: mg/100 mL.

Constituents	Retention time / min	0 y	1 y	2 y	3 y	4 y	5 y	10 y
acetaldehyde	5.665	18.8	11.7	7.1	30.7	26.4	41.8	36.0
ethyl acetate	7.923	191.6	230.1	81.8	82.9	176	218.3	181.9
acetal	8.085	22.3	17.2	9.1	45.5	47.5	74.7	47.5
methyl alcohol	8.552	24.7	33.6	15.6	17.3	26.6	27.6	30.5
ethyl butyrate	12.525	12.8	17	14.3	44.7	56.3	74.3	48.7
sec-butyl alcohol	12.623	2.5	11.8	2.9	8.7	11.4	14.5	6.1
n-propanol	13.146	21.5	34.5	27.7	17.7	20.5	47.3	30.9
isobutanol	15.635	7.0	6.4	12.5	10.4	10.5	14.4	8.7
ethyl valerate	16.829	4.0	11	5.2	15.6	15.8	24.6	11.5
2-amyl alcohol	17.072	1.0	2.7	1.4	1.8	2.7	3.5	2.6
n-butyl alcohol	18.299	20.8	45.5	40.4	9.9	12.4	15	22.4
n-amyl acetate	18.887	17.3	16.3	17.6	17.3	17.3	17.3	17.3
isoamyl alcohol	21.401	11.3	12.4	29.4	14.4	18.7	29.8	23.7
ethyl caproate	21.812	79.9	256.3	224.5	412.5	437.7	686.6	324
n-amyl alcohol	23.223	2.0	3.4	2	1.1	1.3	1.4	2.1
ethyl lactate	27.872	133.4	197.7	231.7	149.5	209.7	128	161.9
hexyl alcohol	28.565	12.3	21.7	16.5	6.6	7.9	9.6	15.6
acetic acid	32.132	105.3	111.7	71.9	69.2	62.5	68.1	70.2
propionic acid	34.997	1.3	1.26	1.7	1	1.1	2.7	2.5
isobutyric acid	35.837	1.5	1.7	1.4	1.2	1.4	1.4	1.9
n-butyric acid	37.488	3.0	17.5	12.5	26	22.5	24.7	31.6
isovaleric acid	38.574	2.3	2.4	1.5	2.3	1.5	1.5	2.7
n-pentanoic acid	40.284	3.2	4.3	2.3	6	4.6	6.6	5.6
n-hexylic acid	42.679	42.2	58.5	59.7	106.7	92.5	142.3	120.6

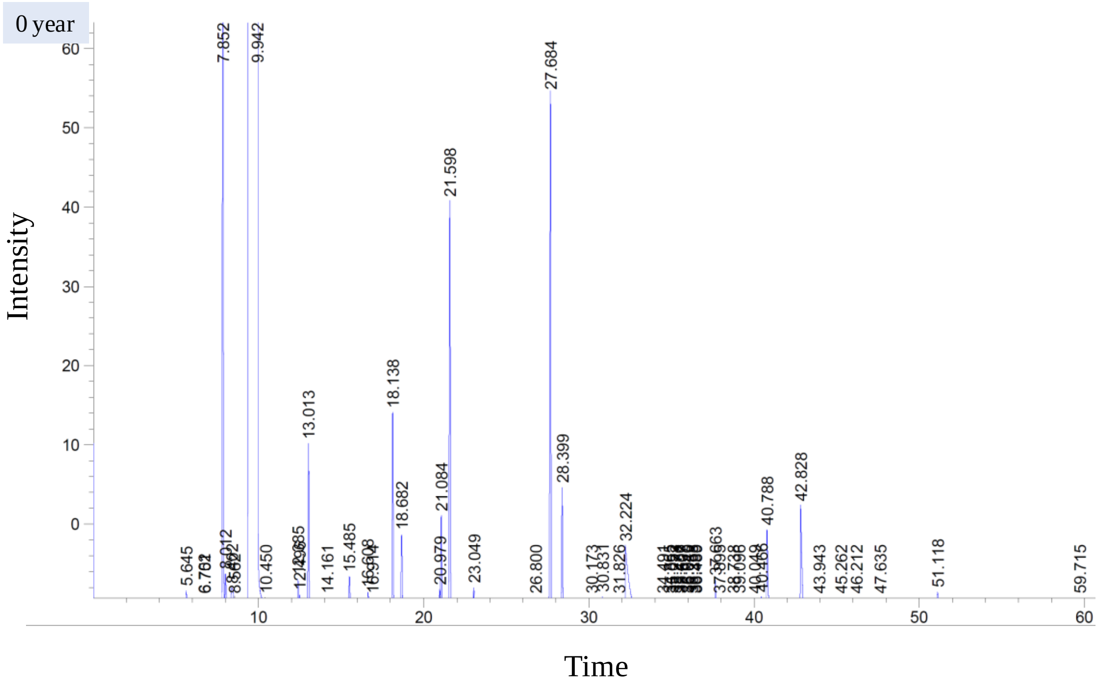

Figure S1. GC Chromatogram of the liquor sample with the aging time of 0 year.

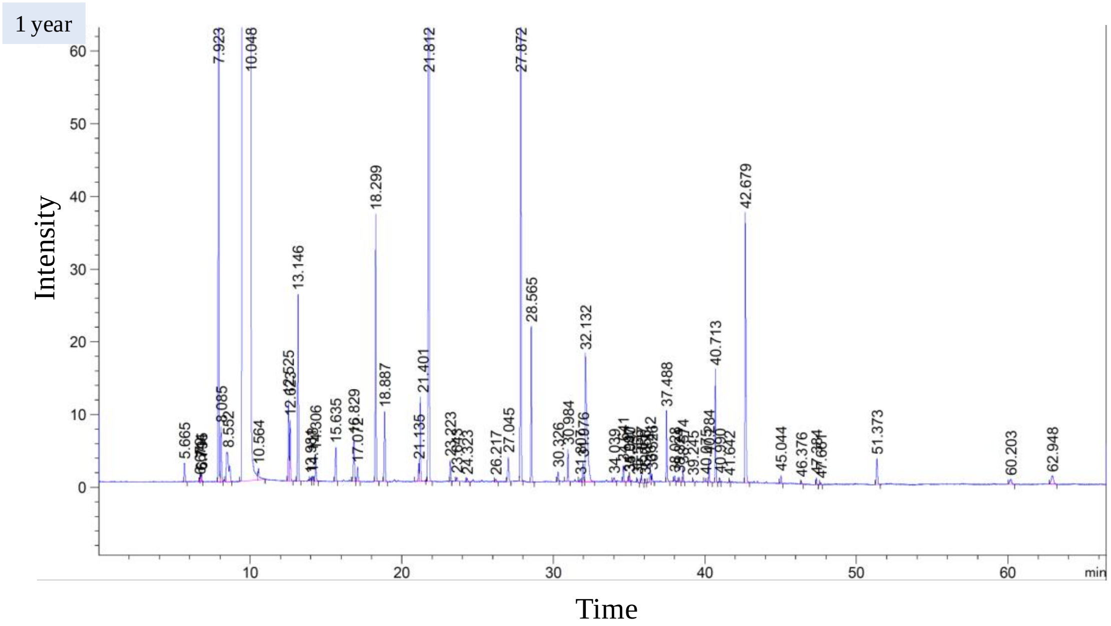

Figure S2. GC Chromatogram of the liquor sample with the aging time of 1 year.

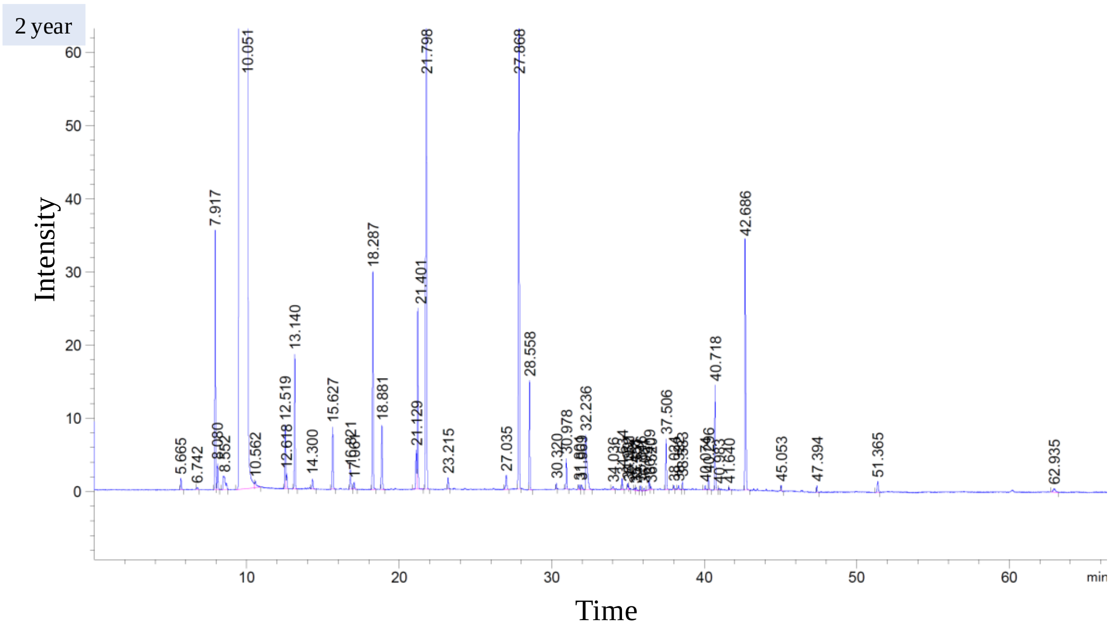

Figure S3. GC Chromatogram of the liquor sample with the aging time of 2 years.

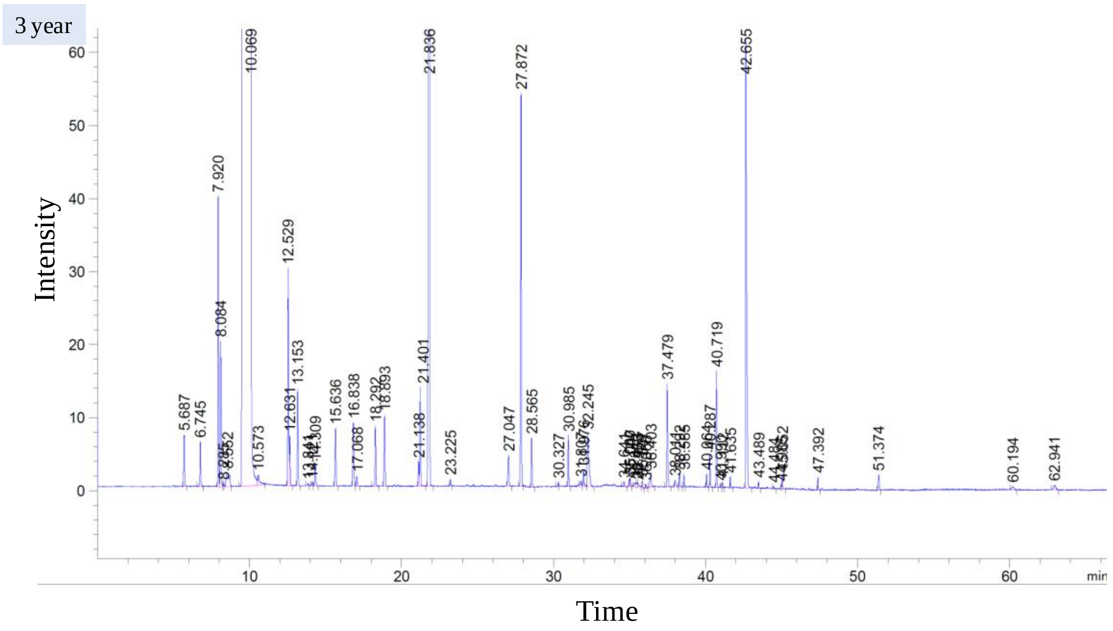

Figure S4. GC Chromatogram of the liquor sample with the aging time of 3 years.

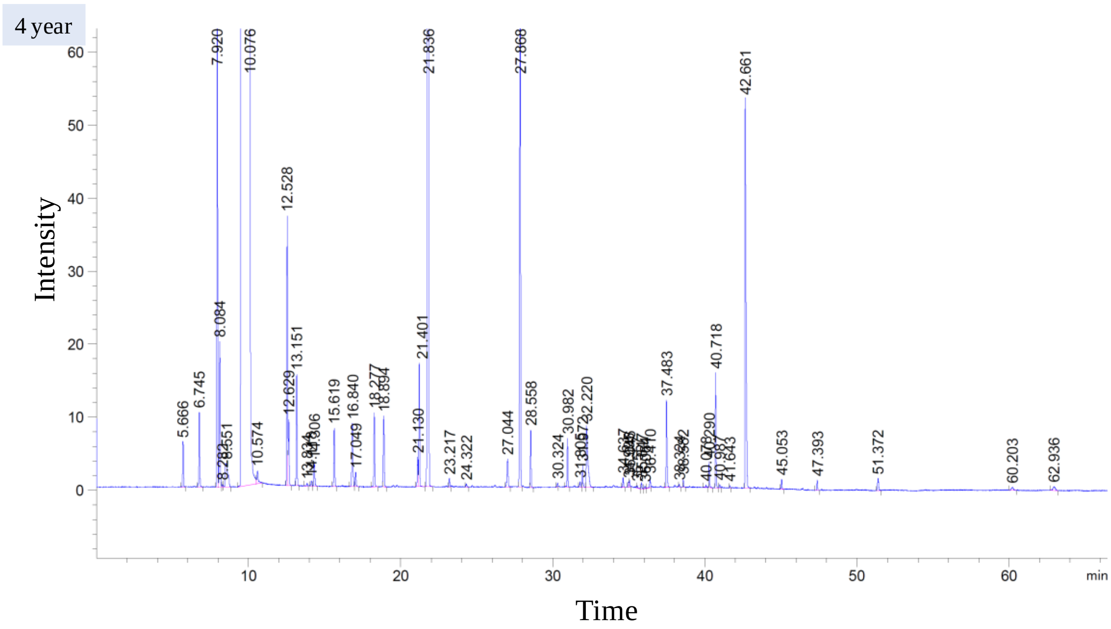

Figure S5. GC Chromatogram of the liquor sample with the aging time of 4 years.

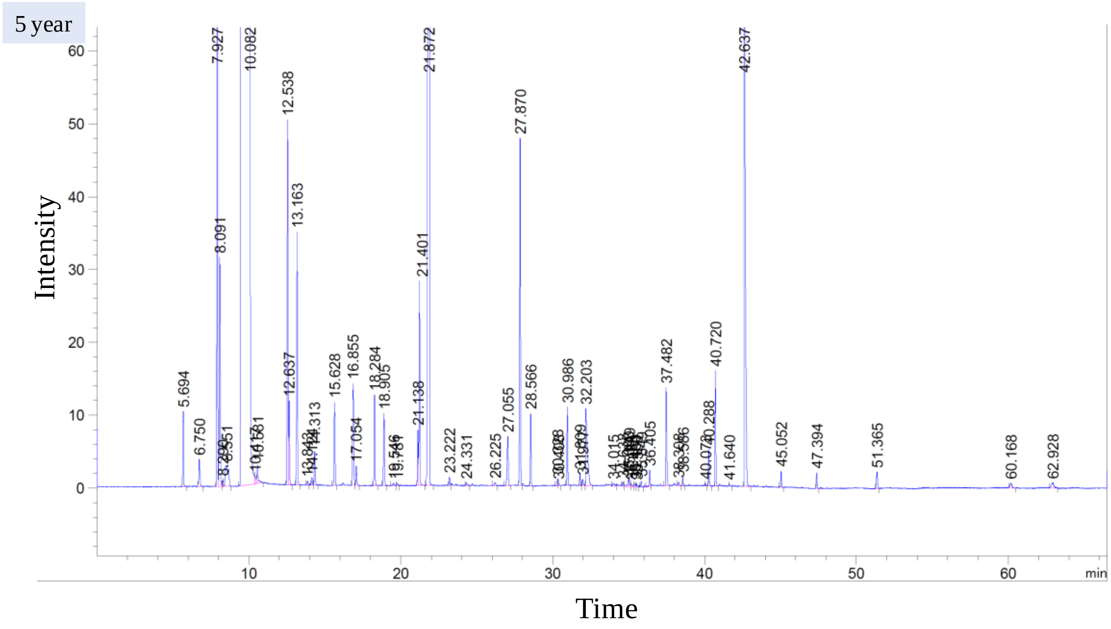

Figure S6. GC Chromatogram of the liquor sample with the aging time of 5 years.

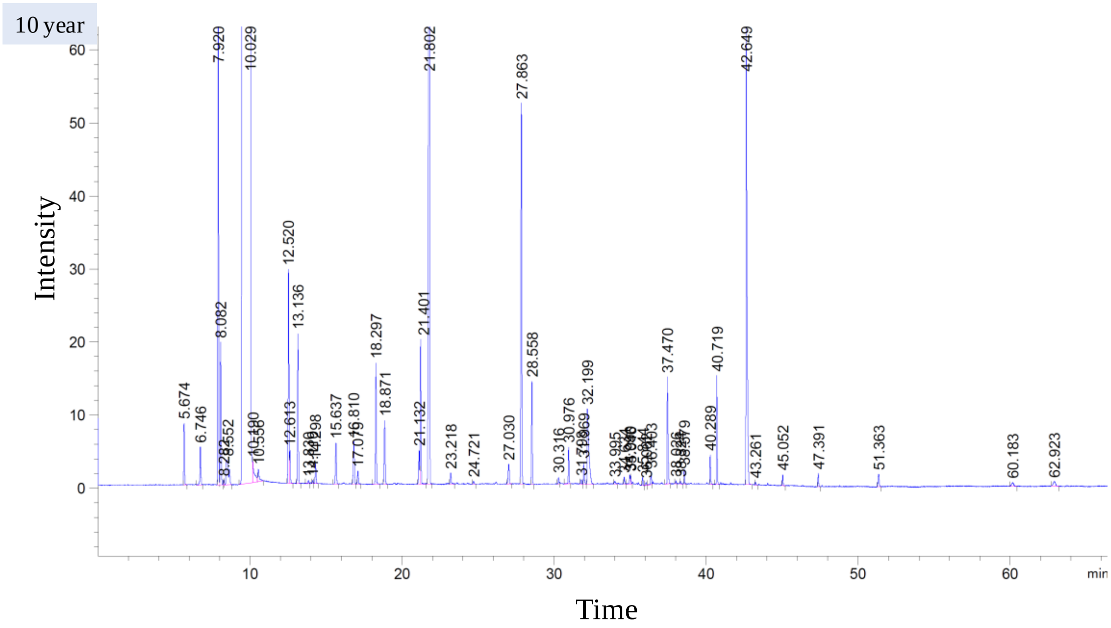

Figure S7. GC Chromatogram of the liquor sample with the aging time of 10 years.
